# Targeted Next-Generation Sequencing Identifies a Recurrent Mutation in *MCPH1* Associating with Hereditary Breast Cancer Susceptibility

**DOI:** 10.1371/journal.pgen.1005816

**Published:** 2016-01-28

**Authors:** Tuomo Mantere, Robert Winqvist, Saila Kauppila, Mervi Grip, Arja Jukkola-Vuorinen, Anna Tervasmäki, Katrin Rapakko, Katri Pylkäs

**Affiliations:** 1 Laboratory of Cancer Genetics and Tumor Biology, Cancer and Translational Medicine Research Unit and Biocenter Oulu, Northern Finland Laboratory Centre NordLab Oulu, University of Oulu, Oulu, Finland; 2 Department of Pathology, Oulu University Hospital and University of Oulu, Oulu, Finland; 3 Department of Surgery, Oulu University Hospital and University of Oulu, Oulu, Finland; 4 Department of Oncology, Oulu University Hospital and University of Oulu, Oulu, Finland; 5 Laboratory of Genetics, Northern Finland Laboratory Centre NordLab Oulu, Oulu, Finland; Cleveland Clinic Genomic Medicine Institute, UNITED STATES

## Abstract

Breast cancer is strongly influenced by hereditary risk factors, a majority of which still remain unknown. Here, we performed a targeted next-generation sequencing of 796 genes implicated in DNA repair in 189 Finnish breast cancer cases with indication of hereditary disease susceptibility and focused the analysis on protein truncating mutations. A recurrent heterozygous mutation (c.904_916del, p.Arg304ValfsTer3) was identified in early DNA damage response gene, *MCPH1*, significantly associating with breast cancer susceptibility both in familial (5/145, 3.4%, *P* = 0.003, OR 8.3) and unselected cases (16/1150, 1.4%, *P* = 0.016, OR 3.3). A total of 21 mutation positive families were identified, of which one-third exhibited also brain tumors and/or sarcomas (*P* = 0.0007). Mutation carriers exhibited significant increase in genomic instability assessed by cytogenetic analysis for spontaneous chromosomal rearrangements in peripheral blood lymphocytes (*P* = 0.0007), suggesting an effect for MCPH1 haploinsufficiency on cancer susceptibility. Furthermore, 40% of the mutation carrier tumors exhibited loss of the wild-type allele. These findings collectively provide strong evidence for *MCHP1* being a novel breast cancer susceptibility gene, which warrants further investigations in other populations.

## Author Summary

Although the contribution of hereditary susceptibility to breast cancer is well-established, the majority of predisposing factors still remain unidentified. Here, we have taken advantage of recent technical and methodological advances and performed a massive parallel sequencing of hundreds of DNA damage response genes in breast cancer cases with indication of hereditary disease susceptibility. We identify a recurrent breast cancer predisposing mutation in *MCHP1* gene. The genetic data combined with the evidence of genomic instability related to identified *MCPH1* mutation and also loss of the other functional gene copy in several mutation carrier tumors establish *MCHP1* as a novel breast cancer susceptibility gene. This provides further tools for the clinical risk assessment of individuals with family burden of breast cancer. Our results reinforce the essential involvement of DNA damage response pathway in prevention of malignancy and indicate that parallel sequencing of the genes from this pathway provides an excellent approach for the identification of novel rare inherited mutations predisposing to this common disease.

## Introduction

Breast cancer is the most common malignancy among women, and the contribution of hereditary susceptibility to its development has been well recognized. Numerous breast cancer susceptibility genes are involved in the DNA damage response, strongly indicating that certain pathways of DNA repair and checkpoint control are necessary for preventing malignancy, particularly in breast epithelial cells. These susceptibility genes, including the major ones *BRCA1* and *BRCA2* along with *PALB2*, are all characterized by several rare, loss-of-function mutations, usually protein truncations. These heterozygous germline mutations predispose carrier individuals to breast, but to some extent also to ovarian cancer [1]. However, as the currently known moderate-to-high risk genes explain only 30% of the familial and 5% of the total breast cancer incidence [1–3], the identification of new genetic susceptibility factors and understanding of their contribution to disease onset is imperative. For this purpose we have performed a targeted next-generation sequencing of altogether 796 genes involved in diverse DNA repair signaling pathways in individuals with indication of hereditary disease susceptibility, originating from the genetically isolated Northern Finnish population. Based on their strong prior evidence for breast cancer association, the analysis was focused on protein truncating mutations, which were evaluated for cancer association by case-control comparisons. This initial sequencing revealed a deletion in the *MCPH1* gene, encoding an early DNA damage response protein. We show here that this recurrent mutation significantly associates with breast cancer susceptibility, and that MCPH1 has an integral role in the maintenance of genomic instability and acts as a tumor suppressor in breast cancer.

## Results

The targeted next-generation sequencing revealed a recurrent deletion in the *MCPH1* gene (also known as *BRIT1*, NM_024596.3: c.904_916del, rs747489687) in 3/189 of the analyzed patients, these carriers being negative for any other known breast cancer associated gene mutations. All observed PTVs and their corresponding frequency in ExAC database (http://exac.broadinstitute.org/) are summarized in **S1 Table**. The *MCPH1* c.904_916del mutation results in a frameshift and premature translation stop (p.Arg304ValfsTer3). In total, the *MCPH1* c.904_916del allele was genotyped in 1370 breast cancer cases (145 familial cases, 75 young cases diagnosed below the age of 40 years, and 1150 cases unselected for a family history of cancer or age at disease onset) and 1160 healthy geographically matched controls (**Table 1**). The highest prevalence for *MCPH1* c.904_916del was observed among the familial cases (5/145, 3.4%), whereas only 5 of the 1160 healthy controls (0.4%) carried the mutation (*P* = 0.003, OR 8.3, 95% CI 2.4–28.9). The association with breast cancer was replicated with the unselected breast cancer cohort, where 16 additional *MCPH1* c.904_916del carriers were identified (16/1150, 1.4%, *P* = 0.016, OR 3.3, 95% CI 1.2–8.9). The average age at disease onset for all mutation carriers was 59 years (variation 27–90 years), which did not differ from the mean in the unselected cohort (58 years, variation 28–93 years). In line with this observation, no mutation carriers were identified in the cohort of young breast cancer cases (0/75).

**Table 1:** Frequency of the heterozygous *MCPH1* c.904_916del mutation among familial, unselected and young breast cancer patients, and in population controls.

Study cohort	N	*MCPH1* c.904_916del (%)	OR^a^	95% CI	*P*-value^b^
Familial BC ^c^	145	5 (3.4%)	8.3	2.4–28.9	0.003
Unselected BC	1150	16 (1.4%)	3.3	1.2–8.9	0.016
Young BC ^c^	75	ND	-	-	1.000
All BC	1370	21 (1.5%)	3.6	1.4–9.6	0.009
Controls	1160	5 (0.4%)	-	-	-

^a^ versus controls

^b^ Fisher´s exact or Pearson’s Chi-Square test

^c^ Includes cases from the initial NGS

BC: breast cancer, CI: confidence interval, ND: not detected, OR: odds ratio

*MCPH1* c.904_916del resides within a common haplotype, suggesting that it is a founder mutation (**S2 Table**). It has also been reposited in ExAC, where its frequency in Finnish population (not stratified for any cancer phenotypes) equals to that observed in our control cohort (16/3305, 0.48% and 5/1160, 0.43%, respectively). When comparing *MCPH1* c.904_916del allele’s frequency in the currently analyzed breast cancer cohort to that reported in ExAC Finns, the statistical evidence for cancer association is even more significant: for familial cohort *P* = 0.001 (OR 7.3, 95% CI 2.7–20.3), for unselected cohort *P =* 0.003 (OR 2.9, 95% CI 1.4–5.8), and for all breast cancer cases *P* = 0.0004 (OR 3.2 and 95% CI 1.7–6.2). Curiously, *MCPH1* c.904_916del is also reported in non-Finnish cohorts in ExAC, although at much lower frequency (3/33 277 in non-Finnish Europeans, and 1/446 in “other” cohort of unknown origin).

In order to exclude the presence of any other known cancer predisposing gene mutations in *MCPH1* c.904_916del carriers, all 21 index cases were analyzed using TruSight One Sequencing panel (Illumina). In 20 of the analyzed patients, *MCPH1* c.904_916del was the only deleterious mutation identified, whereas one unselected case (BR-0336, diagnosed with breast cancer at the age of 79 and melanoma at the age of 82, **Table 2**) carried also pathogenic mutation (rs80357571) in *BRCA1*. In this one case, the disease predisposition can be caused by either *MCPH1* or *BRCA1* mutation, or by their combined effect. However, for the majority (20/21) of *MCPH1* c.904_916del carriers the possibility that other known cancer predisposition mutations could explain their cancer phenotype was excluded.

**Table 2:** Family history of cancers of *MCPH1* c.904_916del positive index cases^a^.

Index ID—cancers/tumors (age at diagnosis)	Breast/ovarian cancer(s) in 1st and 2nd degree relatives (age at diagnosis)	Brain tumors/sarcomas in 1st and 2nd degree relatives (age at diagnosis)	Lung cancer(s) in 1st and 2nd degree relatives (age at diagnosis)	Other cancers (No. of cases) in 1st and 2nd degree relatives
BR-0705 –Br (66)*	Br (66, u, u)	Sar (16)	Lu (61)	Lym (1)
BR-0727 –Br (78)	Br (62, u)	Sar (60)	Lu (25, 49, u)	Col (1), Csu (1)
BR-11124 –Br (59)	Br (95, u)	Bt (Ast) (74^b^)	Lu (58 [+])	Lym (1)
BR-13109 –Br (79)	Br (65)	-	Lu (50)	-
BR-0653 –Br (37)*	Br (49 [+]), Ov (47 [+], u)^c^	-	-	Col (2), Bas (1), Lar (1), Csu (1)
BR-0154 –Br (43)*	Br (45 [+], 59)	-	-	Vul (1)
BR-0884 –Br (54)	Br (58 [+])	-	-	-
BR-0361 –Br (46)*	Br (55, 65)	-	-	-
BR-13145 –Br (51)	Br (u)	-	-	-
BR-1046 –Br (68)	Br (u)	-	-	Sto (1 [+])
BR-0336 ^i^ –Br (79), Mel (82)	Ov (39)	-	-	Liv (1), Csu (1)
97–756 –Bil Br (27/43)*	-	Bt (Men) (49), Sar (4/10^d^)	Lu (72, 50)	Col (1), Sto (1)
BR-0887 –Br (62)	-	Bt (uh) (63 [+])	-	Bas (1 [+]^e^)
BR-1305 –Br (62), Bt (61, 62)	-	Bt (Men) (index [+])	-	Kid (1)
BR-0989 –Br (48)	-	-	Lu (53^f^)	Es (1^f^), Ut (1)
BR-0161 –Br (48)	-	-	Lu (84 [+]^g^)	-
BR-0629 –Br (56)	-	-	Lu (63, 90^h^)	Pan (1), Lar (1), Csu (1)
BR-035 –Br (72)	-	-	-	Pro (1), Or (2), Es (1)
BR-0889 –Br (56)	-	-	-	Sto (1 [+])
BR-0314 –Br (90)	-	-	-	Ut (1)
BR-1036 –Br (60)	-	-	-	-

Ast: astrocytoma, Bas: basalioma, Bt: brain tumor, Br: breast cancer, Bil Br: bilateral breast cancer, Col: colon cancer, Csu: cancer site unknown, Es: esophageal cancer, Kid: kidney cancer, Lar: laryngeal cancer, Liv: liver cancer, Lu: lung cancer, Lym: lymphoma, Mel: melanoma, Men: meningioma, Or: oral cancer, Ov: ovarian cancer, Pan: pancreatic cancer, Pro: prostate cancer, Sar: sarcoma, Sto: stomach cancer, u: age at diagnosis unknown, uh: unknown histology, Ut: uterine cancer, Vul: vulvar cancer

[+] tested *MCPH1* c.901-921del positive

*Familial cohort

^a^ All the tumors of known non-carriers have been excluded; only individuals potentially having *MCPH1* mutation are listed

^b^ Basalioma also present

^c^ Ovarian tumors

^d^ Rhabdomyosarcoma at the age of 4 and chondrosarcoma at the age of 10

^e^ 3^rd^ degree relative of the index; included based on the positive mutation status

^f^ Stomach cancer also present

^g^ Prostate cancer also present

^h^ Kidney cancer also present

^i^ Heterozygous also for pathogenic *BRCA1* mutation rs80357571

**Fig 1:**
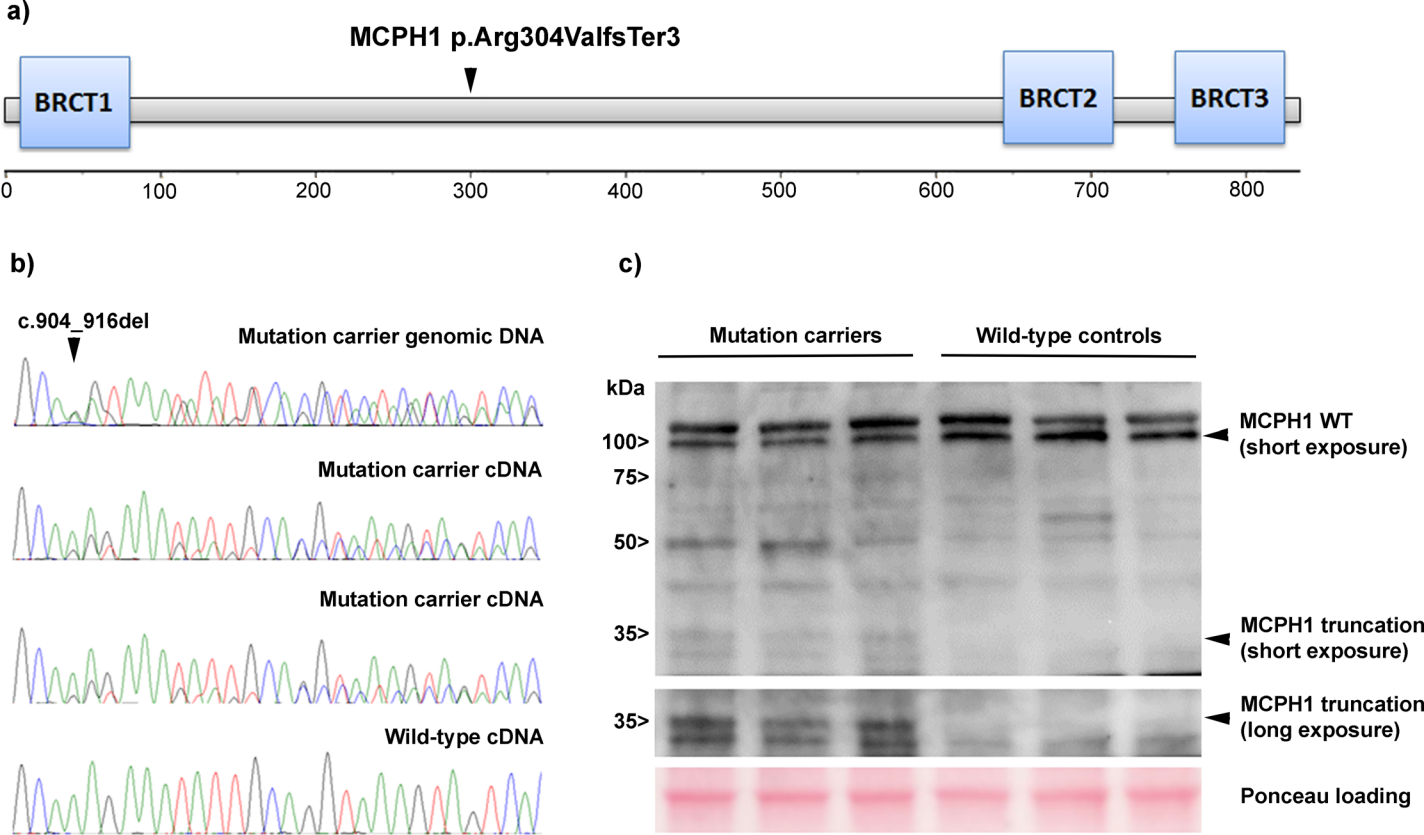
Effect of *MCPH1* c.904_916del mutation at the mRNA and protein level. (**A**) Schematic presentation of the MCPH1 protein and the position of the observed truncating mutation. (**B**) Sequence chromatogram comparisons of genomic DNA and cDNA of heterozygous mutation carriers and a wild-type control. (**C**) Immunoblotting of 3 mutation carriers and 3 non-carriers with an antibody directed towards the amino-terminus of MCPH1. The representative image of altogether three independent experiments is shown.

*MCPH1* encodes an important early DNA damage response protein. Following DNA damage, MCPH1 is rapidly recruited to nuclear foci along with MDC1, 53BP1 and γH2AX. It is responsible for the recruitment of several other integral DNA repair proteins, including ATM, NBS1, BRCA2 and RAD51 to the site of damage [4,5]. The multifunctionality of this protein is further emphasized by reports providing a role for MCPH1 as an important link between chromatin remodeling and DNA damage response. The loss of MCPH1 causes impaired chromatin relaxation, resulting from decreased association of the ATP-dependent chromatin remodeling complex SWI-SNF with chromatin. This might provide the explanation for its crucial role in the recruitment of repair proteins to DNA lesion [6]. Reflecting these functions, biallelic *MCPH1* mutations result in microcephaly and premature chromosome condensation syndrome (MIM #251200). At cellular level, these patients show genomic instability and defective DNA repair [4,7,8]. Analogous to many other proteins crucial for the DNA damage response, MCPH1 contains BRCA1 COOH terminal (BRCT) [9] domains: the N-terminal BRCT is essential for the rescuing of premature chromosome condensation in MCPH1-deficient cells [10], whereas the two C-terminal BRCTs are necessary for ionizing radiation induced foci formation and DNA damage response [4,11]. The currently observed deletion abolishes both C-terminal BRCT domains (**Fig 1A**), and is predicted to cause defective function in DNA damage response as modeled previously [4]. As expected, the amount of full-length protein was reduced to 52% in the studied patient-derived heterozygous *MCPH1* c.904_916del lymphoblastoid cell lines (**Fig 1C**). Curiously, the mutant transcript was, to some extent, able to escape nonsense-mediated decay as demonstrated by cDNA specific sequencing (**Fig 1B**). In addition, a faint band marking the truncated protein of the expected 35 kDa in size was observed in immunoblotting (**Fig 1C**), indicating a partial stability of the truncation product.

**Fig 2:**
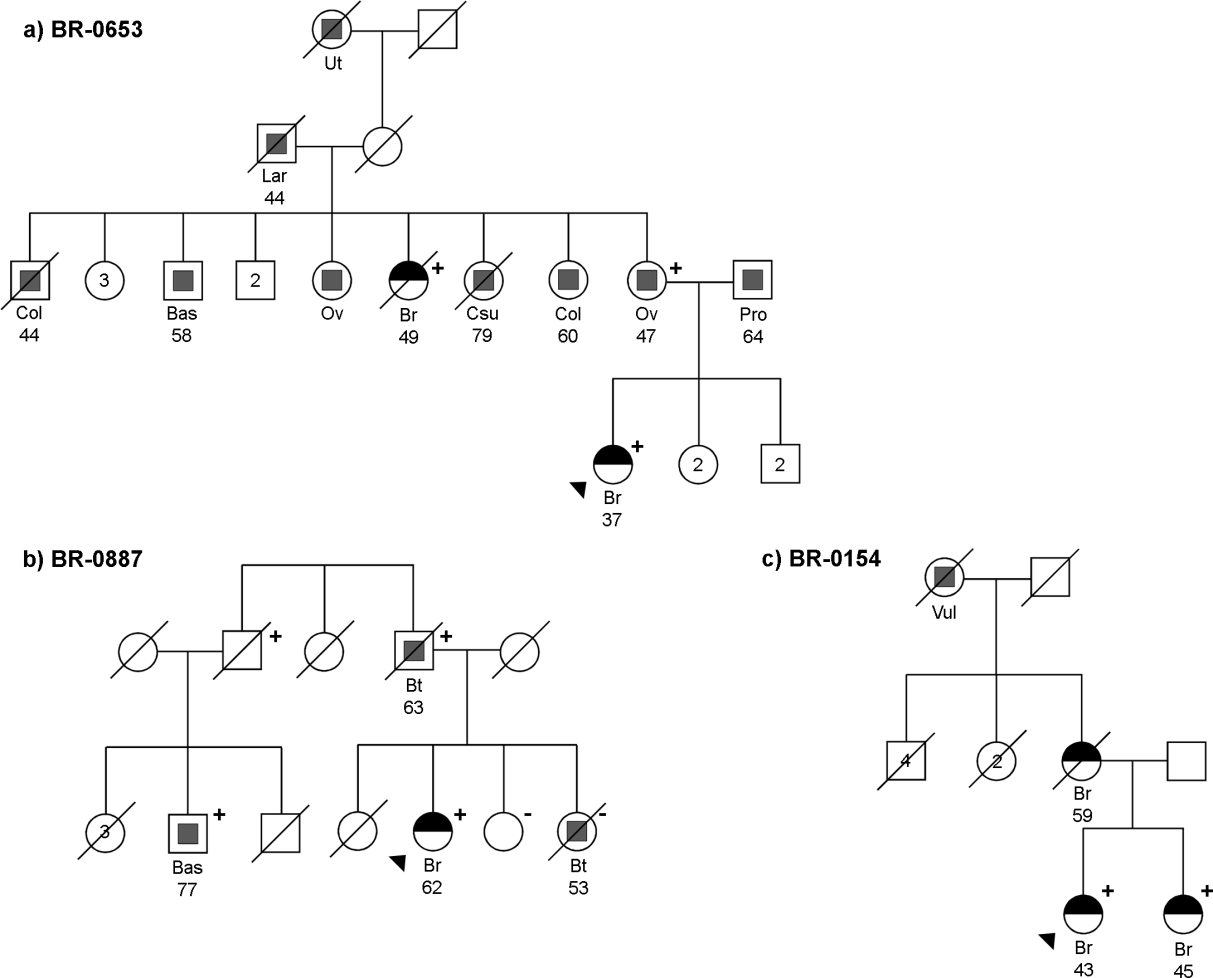
Examples of *MCPH1* mutation positive families (A-C). Patients with breast cancer are marked with black half circles. Other cancer types are marked with grey squares. The age at diagnosis, when known, is marked below the cancer type. Individuals genotyped for *MCPH1* c.904_916del are marked with either a plus (mutation positive) or a minus sign (mutation negative). A slashed pedigree symbol indicates a deceased individual. Triangle indicates the initially studied index patient (BR-0653, BR-0887 and BR-0154, respectively). Abbreviations: Bas: basalioma, Bt: brain tumor, Br: breast cancer, Col: colon cancer, Csu: cancer site unknown, Lar: laryngeal cancer, Ov: ovarian tumor, Pro: prostate cancer, Ut: uterine cancer, Vul: vulvar cancer.

All available information and additional DNA samples from the c.904_916del mutation carrier families were used to study the potential segregation of the mutation with cancer phenotype (**Fig 2** and **Table 2**). In support of inherited susceptibility to the disease, 44% (7/16) of the 16 *MCPH1* mutation carriers from the unselected cohort also had at least one breast and/or ovarian cancer case among their 1st and 2nd degree relatives. Besides initially studied index cases, four relatives with breast cancer were available for mutation testing. Of these three were positive for the *MCPH1* c.904_916del mutation. The relatives of *MCPH1* c.904_916del carriers were also reported to have several other types of malignancies, the most common being lung cancer, which occurred altogether in 38% (8/21) of all carrier families. Notably, some of these lung cancers were diagnosed at very young age (25 years) and in confirmed non-smokers. Also brain tumors and/or sarcomas were overrepresented in the carrier families (28.6%, **Table 2**); the difference being highly significant when compared to the incidence of these cancers in the analyzed cohorts (5.1%, *P* = 0.0007, OR 7.4 95% CI 2.7–19.9). One of the initially analyzed breast cancer cases was even diagnosed with two brain tumors, meningiomas. The association between breast cancer and meningiomas has previously been reported [12], suggesting shared genetic and/or environmental risk factors. The tumor spectrum of breast cancer, brain tumor, sarcoma and to some extent also lung cancer is reminiscent to that of Li-Fraumeni syndrome (LFS), caused by germline mutations in *TP53*, although meningiomas are only rarely reported in LFS patients [13]. The similar tumor spectrum might be explained by a recent report providing a role for MCPH1 as a regulator of p53 stability by blocking MDM2-mediated p53 ubiquitination [14].

Given the numerous functions of MCPH1 in DNA damage response, we determined the impact of c.904_916del to genomic stability assessed by chromosomal analysis [15] in non-transformed peripheral blood lymphocytes of 7 mutation carriers negative for other known breast cancer associated mutations. Samples from nine non-carrier individuals were used as controls. Based on epidemiological studies people with elevated frequency of chromosomal aberrations in their peripheral blood lymphocytes have a significantly elevated risk of developing cancer, reflecting either early biological effects of genotoxic carcinogens or individual’s inherited cancer susceptibility [16]. The *MCPH1* c.904_916del carriers exhibited a significantly increased frequency of spontaneous chromosomal rearrangements (*P* = 0.0007) when compared to controls (**Table 3** and **S1 Fig**). All observed chromosomal aberrations were considered random, as no preference for specific break site or evidence for clonality was observed. This suggests an effect for *MCPH1* c.904_916del mutation already in the heterozygous state. The prevalence of increased incidence of chromosomal rearrangements in *MCPH1* carriers is consistent with the increased genome instability caused by defective DNA damage response genes [15,17,18]. Total depletion of MCPH1 has also been reported to lead to chromosomal aberrations in mammary epithelial cells [4], and experiments in mice show that MCPH1 deficiency results in increased chromosomal aberrations and promotes long-latency tumor formation [19].

**Table 3:** Occurrence of chromosomal aberrations in blood lymphocyte metaphases of heterozygous *MCPH1* c.904_916del mutation carriers and healthy controls.

Type of aberration	Median of chromosomal aberrations observed per 100 metaphases (min–max)		*P*-value^a^
	Carriers (n = 7)	Controls (n = 9)	
Telomeric associations	0.0 (0.0–2.0)	ND	0.4370
Chromatid/chromosome breaks, deletions	1.9 (0.0–4.0)	1.8 (0.0–4.0)	0.7190
Simple chromosomal rearrangements^b^	4.0 (1.9–8.0)	0.0 (0.0–2.0)	0.0010
Complex chromosomal rearrangements^c^	0.0 (0.0–3.8)	ND	0.1750
Total rearrangements (simple+complex)	5.7 (2.0–10.0)	0.0 (0.0–2.0)	0.0007

^a^ Mann-Whitney *U*-test

^b^ Inversions, ring chromosomes, translocations (≤3 break rearrangements)

^c^ Translocations (≥4 break rearrangements) and marker chromosomes

ND: not detected

Loss of heterozygosity (LOH) analysis at the *MCPH1* locus demonstrated that the wild-type allele was lost in 40% (8/20) of the studied c.904_916del carrier breast tumors (**S3 Table** and **S2 Fig**). This indicates tumor suppression according to Knudson´s two hit model, analogous to that of *BRCA1*/*BRCA2* mutation carrier tumors [20]. Although *MCPH1* resides in chromosomal region 8p23.1, frequently found aberrated in different malignancies including lung, ovarian and breast cancer [21–23], only one of the studied tumors had lost the mutant allele. This provides evidence for the observed LOH being directional rather than a random event. In line with this, 2/4 studied non-breast tumors (lung carcinoma and liver metastasis from gastric adenocarcinoma) of *MCPH1* c.904_916del carriers had lost the wild-type allele. The *MCHP1* mutation carrier breast tumors (*n* = 22, **S3 Table**) also showed a trend towards triple-negativity (i.e. ER-, PR-, HER2-; 28% of the tumors, *p* = 0.07), but larger cohorts would be needed to confirm any significant associations.

Based on the observation of LOH in the tumors and the role of MCPH1 in DNA DSB repair, we tested whether malignant (MCF7) mammary epithelial cells depleted in MCPH1 by transient RNA-interference are sensitive to poly (ADP-ribose) polymerase (PARP) inhibitors, similarly to BRCA1 and BRCA2 depletion [24]. Our results show only a modest sensitization to PARP inhibition and these results were replicated using MCF10A cells (**S3 Fig**). This indicates that the role of MCPH1 in DNA damage response differs somewhat from those directly involved in DSB repair via homologous recombination. Thus, *MCPH1* mutation carriers might benefit from treatment with PARPi only when combined with some other treatments, which should be tested further.

## Discussion

Current study provides strong genetic evidence for the association of *MCPH1* c.904_916del heterozygosity with inherited breast cancer predisposition, and adds yet another link to human disease for the multifunctional MCPH1 protein. In the currently studied cohorts the *MCPH1* c.904_916del mutation explains 3.4% of the familial and 1.4% of the unselected cases, these frequencies being comparable to that observed for Finnish *PALB2* c.1592delT founder mutation [3]. Although c.904_916del allele’s frequency in familial cohort is lower than the frequency of *BRCA1* and *BRCA2* mutations, which collectively explain 20% of the Finnish breast and ovarian cancer families [25,26], in unselected cases the combined frequency of different *BRCA1/2* mutations (1.8%) [27] is roughly the same as observed for this single *MCPH1* mutation. Based on case-control comparisons, the *MCPH1* c.904_916del carriers have a comparable breast cancer risk to that observed for the Finnish *PALB2* c.1592delT carriers, evaluated by using both unselected breast cancer (3.3-fold risk for *MCPH1* and 3.9-fold risk for *PALB2*) and familial breast cancer cohort (8.3-fold risk for *MCPH1* and 11.3-fold risk for *PALB2*) [3], the risk being at least moderate and potentially high. *PALB2* has recently been reported to confer a breast cancer risk comparable to that of *BRCA2* mutations, indicating the need to include it in the clinical diagnostics along with *BRCA1* and *BRCA2* mutation testing [28]. As MCPH1 is also important for brain development, demonstrated by the fact that inherited biallelic *MCPH1* mutations result in microcephaly, it is of note that many of the currently identified *MCPH1* c.904_916del carrier families also exhibited brain tumors.

The importance of MCPH1 for prevention of malignancy, particularly in breast epithelial cells, has been indicated by previous studies reporting somatic MCPH1 downregulation in multiple breast cancer cell lines and breast cancer specimens [4,29]. *MCPH1* alterations have also been reported in TCGA datasets retrieved from cBioPortal Cancer Genomics database [30], not only in breast tumors (about 7%) but also in other malignancy types observed in the currently identified carrier families (sarcomas 3.5%, brain tumors 1% and lung cancer 8%). The current genetic data brings now firm evidence for the role of *MCPH1* in breast cancer prevention. The effect of *MCPH1* c.904_916del mutation on the maintenance of genomic integrity and cancer predisposition is also supported by the significant increase in chromosomal rearrangements observed in the untransformed cells of the mutation carriers, and the tumor suppressor role of MCPH1 is reinforced by loss of wild-type allele in 40% of the *MCPH1* c.904_916del mutation carrier breast tumors. Altogether, this data indicates that common causal molecular events can occur both in inherited forms and in non-inherited forms of breast cancer. In order to reveal the molecular details linked to this mutation and its role in cancer predisposition more detailed functional characterization using genome editing of breast epithelial cells is required. These subsequent studies on *MCPH1* c.904_916del carriers could serve as a model for somatically MCPH1 deficient cancers as well.

The identification of MCPH1 as a novel breast cancer susceptibility gene further reinforces the essential involvement of compromised DNA DSB response pathway in malignancy development. Thus, targeted high depths of coverage next-generation sequencing of the genes from DSB pathway provides an excellent approach for the identification of novel rare germline mutations predisposing to this common disease. The use of founder populations provides further advantage for this: gene’s contribution to hereditary cancer susceptibility is easier to prove, if many families harboring the same predisposing mutation can be identified. Even though the currently identified *MCPH1* c.904_916del is evidently Finnish founder mutation, ExAC database reports it in other populations as well, although at much lower frequency, thus warranting further investigations. Given the mutation spectrum of *MCPH1* gene reported in ExAC, other population specific breast cancer associated *MCPH1* mutations are also likely to exist. Altogether, the current results collectively provide strong evidence for *MCHP1* being a novel breast cancer susceptibility gene, which will provide further tools for the clinical risk assessment of individuals with family burden of this disease.

## Materials and Methods

### Targeted next-generation sequencing

Agilent HaloPlex 5 Mb custom target enrichment system was used for the initial screening of germline mutations in 189 breast cancer patients with indication of hereditary disease susceptibility [*n* = 62 cases with young disease onset (<40y), and *n* = 127 cases with family history of breast or breast and ovarian cancer]. 16/189 of the sequenced patients carried known mutations in *BRCA1/2*, *PALB2* or *TP53* [3,26,31]. They were included for the validation of the mutation detection sensitivity, and also to identify potential risk modifiers. Target genes (*n* = 796) for the sequencing were selected among those encoding 1) proteins identified as being part of DNA repair processes using the GeneOntology database searches and STRING v.9.0 (*n* = 612) [32,33] and 2) novel BRCA1 and PALB2 interacting proteins identified in protein complex purification assays performed with epitope tagged versions of the proteins in HeLaS3 cells (*n* = 184). Gene list, genomic interval and percentage of gene coverage are presented in **S1 Table**. Sequencing of the enriched target regions (coding regions, splice sites and 5’ and 3’ UTR regions) of selected genes was performed with HiSeq2500 platform (Illumina). The sequencing resulted in mean read depth of 174x per sample for the captured region (total bait of 8.38 Mb). The minimum read depth of 7 was used as a threshold level for the variant calling procedure in the bioinformatics pipeline developed by the Finnish Institute of Molecular Medicine [34]. In total, mean of 90.5% of the captured region was covered at least by 7 reads for the analyzed samples. Annotation and characterization of variants was performed with wANNOVAR [35] and SureCall v.1.0 (Agilent), with the focus on protein truncating mutations. The manual examination and visualization of the sequence data was done using the Integrative Genomics Viewer v.2.3 [36]. Mutations were confirmed with Sanger sequencing (ABI3500xL Genetic Analyzer, Applied Biosystems).

### Mutation genotyping

*MCPH1* c.904_916del was genotyped with High-resolution Melt (HRM) analysis (CFX96, Bio-Rad) in familial, young and unselected breast cancer cases, and in geographically matched population controls. Positive control for *MCPH1* c.904_916del was included in all analyses and samples with differing melting curves were confirmed by Sanger sequencing. The familial cases were affected index cases of 145 Northern Finnish breast, or breast and ovarian cancer families, all negative for known pathogenic *BRCA1/BRCA2/PALB2/TP53* mutations [3,26,31]. Of these, 98 were considered as high risk families with 1) three or more breast and/or ovarian cancers in 1st or 2nd degree relatives, or 2) two cases of breast, or breast and ovarian cancer in 1st or 2nd degree relatives, of which at least one with early disease onset (<35 years), bilateral breast cancer, or multiple primary tumors including breast or ovarian cancer in the same individual. The remaining 47 families were indicative of moderate disease susceptibility, and had two cases of breast cancer in 1st or 2nd degree relatives, of which at least the other was diagnosed below the age of 50. Young breast cancer cohort consisted of 75 Northern Finnish patients, diagnosed with breast cancer at or below the age of 40 (median 38, variation 25–40 years. These patients were unselected for a family history of the disease, and negative for known pathogenic *BRCA1/BRCA2/PALB2* mutations [37]. The unselected cohort consisted of 1150 consecutive breast cancer cases operated at the Oulu University Hospital during 2000–2013. They were unselected for a family history of cancer or age at disease onset. 1160 healthy geographically matched anonymous Northern Finnish Red Cross blood donors (*n* = 704 females and *n* = 456 males) were used as population controls. Only the health status at the time of blood donation was known in addition to birth year and gender. All the analyzed patients and healthy controls were geographically matched, and have given their informed consent. Permission to use the above mentioned patient and control materials for the study has been obtained from the Finnish Ministry of Social Affairs and Health (Dnr 46/07/98), and the Ethical Committee of the Northern-Ostrobothnia Health Care District (Dnr 88/2000+amendment).

### TruSight One Sequencing panel

All identified *MCPH1* mutation carrier index cases were analyzed for the prevalence of additional deleterious germline mutations using TruSight One Sequencing panel. Sequencing of the 4813 genes included in the panel (including 94 genes from TruSight Cancer suspected to play a role in cancer predisposition, but also other important genes such as *RAD50* and currently studied *MCPH1*) was performed with NextSeq550 platform (Illumina). Sequencing resulted in mean read depth of 183x per sample for the captured region (12 Mb). In total, mean of 98.5% of the captured region was covered at least by 10 reads and 92.1% at least by 50 reads for the analyzed samples. Within BaseSpace Onsite Genomics computing environment (Illumina), BWA Enrichment (BWA Genome Aligner Software and the GATK Variant Caller) was used for sequence alignment and variant calling, Illumina VariantStudio for annotation, filtering and classification of the variants, and Intergrative Genomics Viewer for data visualization.

### Loss-of-heterozygosity analysis of carrier tumors

Genomic DNA was extracted from formalin-fixed, paraffin-embedded tumors of *MCPH1* c.904_916del carriers. Loss-of-heterozygosity (LOH) was studied by sequencing of 198 bp segment flanking the mutation site. The comparison of allelic ratios in tumor and corresponding normal DNA was based on the exact peak height values from the sequence chromatograms (ab1Peak Reporter Tool, Applied Biosystems). Allelic imbalance (AI) value was calculated for each sample pair (tumor vs. normal), and those showing AI (values >1.67 or <0.60) were considered as having LOH [38].

### Cell culture

Lymphoblastoid cell lines (LCLs) of 3 *MCPH1* c.904_916del carriers and 3 healthy non-carriers were created by immortalization of peripheral blood B-lymphocytes with Epstein-Barr virus. LCLs were cultured in RPMI 1640 medium (Gibco, Invitrogen) supplemented with 20% fetal bovine serum (FBS), 1% L-glutamine (Gibco, Invitrogen). MCF7 cells were maintained in Dulbecco´s modified eagle medium (Sigma), supplemented with 10% FBS and 1% penicillin/streptomycin.

### Chromosomal analysis

Chromosomal analysis of 7 *MCPH1* c.904_916del carriers and 9 wild-type controls was carried out on metaphases obtained from regular short-term 3-day cultures of peripheral blood T-lymphocytes [15]. The blood samples of the patients selected for chromosomal analysis were collected at least 5 years after the initial breast cancer diagnosis and received treatment. The controls used were healthy, age-matched female individuals. A minimum of 50 Giemsa-banded metaphases for each sample were evaluated by light microscopy and photographed with an automatic chromosome analyzer (CytoVision version 7.2, Applied Imaging). Chromosomal aberrations were divided into five classes: 1) telomeric associations, 2) chromatid/chromosome breaks and deletions, 3) simple chromosomal rearrangements, 4) complex chromosomal rearrangements, and 5) total rearrangements.

### Sequencing of cDNA

Total mRNA was isolated from the LCLs using RNeasy Mini Kit (Qiagen) and reverse transcribed with iScript cDNA synthesis Kit (Bio-Rad). The expression of *MCHP1* wild-type and mutant allele was studied the by cDNA specific sequencing.

### Immunoblotting analysis

For immunoblotting of MCPH1, protein lysates from LCLs of 3 mutation carriers and 3 non-carrier controls were extracted in NETN300 buffer, analyzed by SDS-PAGE using 7.5% mini-PROTEAN TGX gel (Bio-Rad) and transferred onto Immobilon-P PVDF membrane (Millipore). The primary MCPH1 antibody (R&D systems, AF3998) was a goat polyclonal antibody raised against the amino acids 1–250 mapping to the N-terminus of MCPH1 of human origin. Horseradish peroxidase-conjugated antibody (Jackson ImmunoResearch Laboratories, 305-035-045) was used as a secondary antibody. Visualization was done using SuperSignal West Femto chemiluminescent substrate (Thermo Scientific). Results were repeated in three independent experiments with variable loading order of the samples.

### PARP inhibitor sensitivity

SMARTpool siGENOME siRNAs (Dharmacon) was used for *MCPH1* and *BRCA2* gene silencing, siGENOME Non-Targeting siRNA Pool (Dharmacon) was used as a negative control. Cells were transfected using Lipofectamine RNAiMAX (Invitrogen), and were exposed to varying concentrations (0μM–10μM) of olaparib (Selleckchem, AZD2281) for 7 days. The drug and the media were replenished every 3 days. Cell viability was estimated using CellTiter-Glo reagent (Promega) and analyzed by GraphPadPrism v.5.0 (GraphPad Software).

### Statistical analyses

Carrier frequencies between cases and controls were compared using Fisher´s exact or Pearson’s Chi-Square test. Mann-Whitney U-test was used to determine the difference in the number of different chromosomal aberrations between *MCPH1* c.904_916del carriers and controls (IBM SPSS Statistics 20.0 for Windows, SPSS Inc.). All *P*-values were two-sided.

## Supporting Information

S1 Fig Typical chromosomal aberrations seen in peripheral blood lymphocytes of heterozygous *MCPH1* c.904_916del mutation carriers. Metaphases shown in (A) and (B) are derived from different carrier individuals and demonstrate simple chromosomal rearrangements [der(7), der(5), t(6p;7p) and t(6q;7q)].
(TIF)

S2 Fig Loss of heterozygosity analysis chromatograms of three sample pairs (normal vs. tumor). Mutated allele has C (blue) and wild-type allele T (red) at the site indicated by the arrow.
(TIF)

S3 Fig Effect of *MCPH1* silencing on olaparib sensitivity. (A) Percentage of cell survival following olaparib administration in MCF7 cells that had been transfected with the indicated siRNAs. Vertical bars represent the standard error of the mean of four independent experiments. (B) Knockdown efficiency in the transfected MCF7 cells measured by qPCR. Corresponding figures from assays using MCF10A cells (C) and (D).
(TIF)

S1 Table Sequenced genes, their coverage, observed mutations and their corresponding frequencies in ExAC database. (XLSX)

S2 Table Haplotype analysis of *MCPH1* c.904_916del mutation carriers. (XLSX)

S3 Table Breast cancer pathology of *MCPH1* mutation carriers. (XLSX)
