# Supplementary figures and images for: Targeted Next-Generation Sequencing Identifies a Recurrent Mutation in MCPH1 Associating with Hereditary Breast Cancer Susceptibility
Source: PLoS Genet. 2016 Jan 28;12(1):e1005816. doi: 10.1371/journal.pgen.1005816 (PMC4731077; doi:10.1371/journal.pgen.1005816)

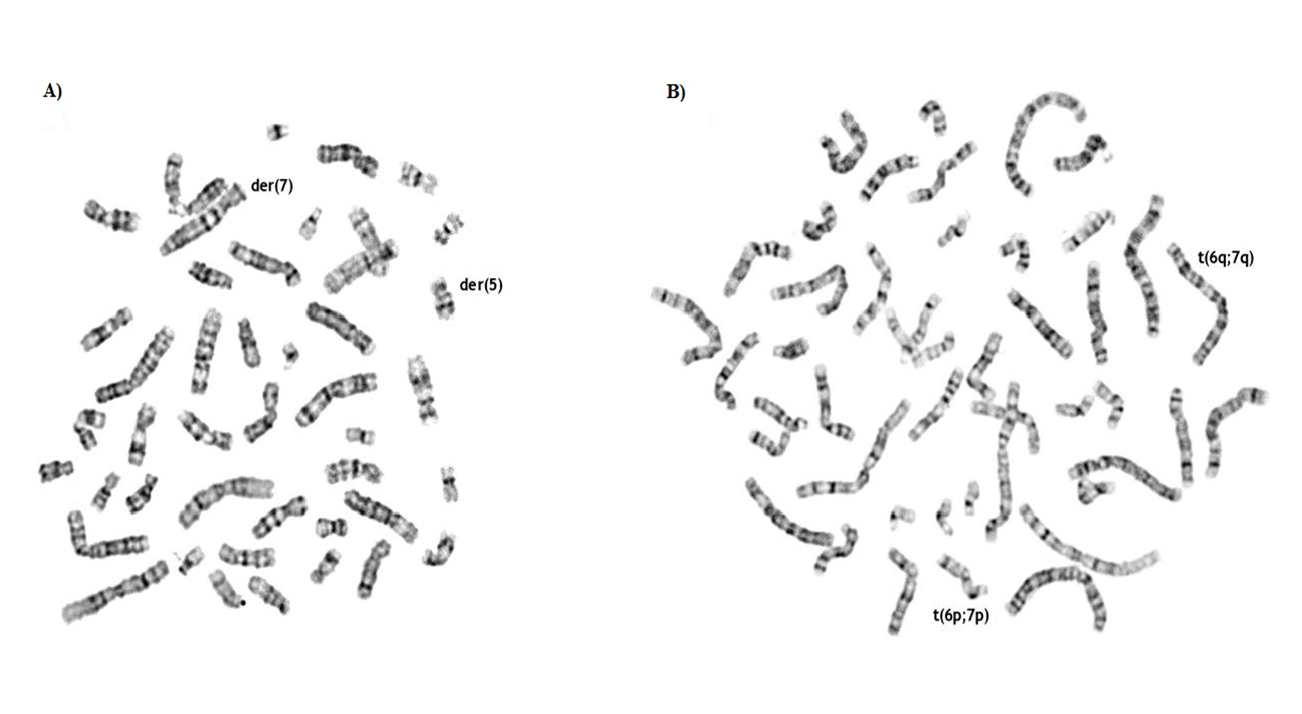

Supplement: S1 Fig — Metaphases shown in (A) and (B) are derived from different carrier individuals and demonstrate simple chromosomal rearrangements [der(7), der(5), t(6p;7p) and t(6q;7q)]. (TIF) [file pgen.1005816.s001.tif]

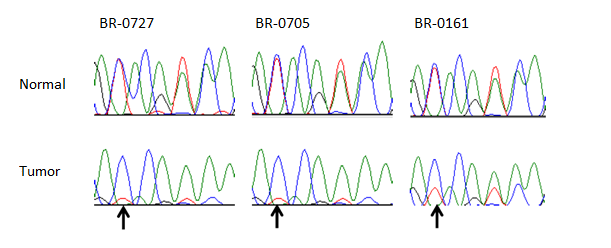

Supplement: S2 Fig — Mutated allele has C (blue) and wild-type allele T (red) at the site indicated by the arrow. (TIF) [file pgen.1005816.s002.tif]

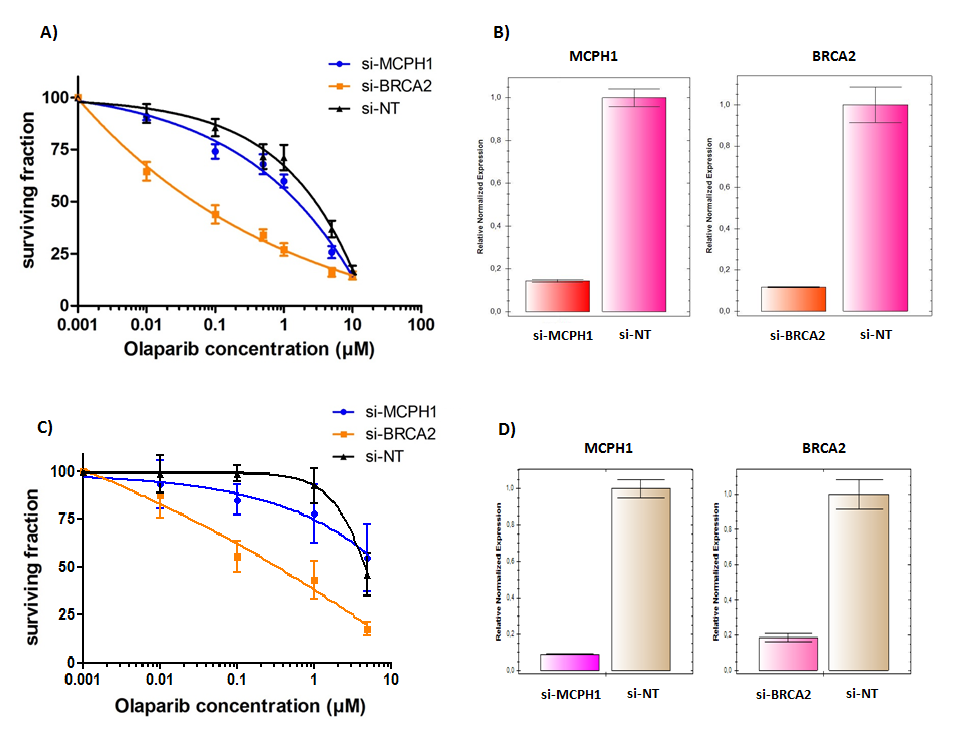

Supplement: S3 Fig — (A) Percentage of cell survival following olaparib administration in MCF7 cells that had been transfected with the indicated siRNAs. Vertical bars represent the standard error of the mean of four independent experiments. (B) Knockdown efficiency in the transfected MCF7 cells measured by qPCR. Corresponding figures from assays using MCF10A cells (C) and (D). (TIF) [file pgen.1005816.s003.tif]
